# Mouse cardiac MRI on a 3 T clinical system using a low cost setup

**DOI:** 10.1186/1532-429X-11-S1-P253

**Published:** 2009-01-28

**Authors:** Timothy F Christian, Jay Gonyea, Stephen P Bell, Trevor Andrews

**Affiliations:** 1 University of Vermont, Burlington, VT USA; 2 Philips Health Care, Burlington, VT USA

**Keywords:** Blood Pool, Endorectal Coil, Inversion Recovery Imaging, Loop Coil, Dual Gradient

## Background

Using a mouse model for cardiac imaging is advantageous due to the wide array of gene alterations possible to investigate a range of disease states. However, the physical challenges for CMRI in mice are significant (small size, high metabolic rate). In order to minimize these issues, many centers use dedicated high field small bore systems with specialized RF coils which are specific to small rodent imaging. Not all centers have the resources or volume to sustain such dedicated magnets but may still require mouse imaging studies for specific questions.

## Purpose

This study describes a lower cost alternative to a complete dedicated system for mouse CMR.

## Methods

**Figure 1:**
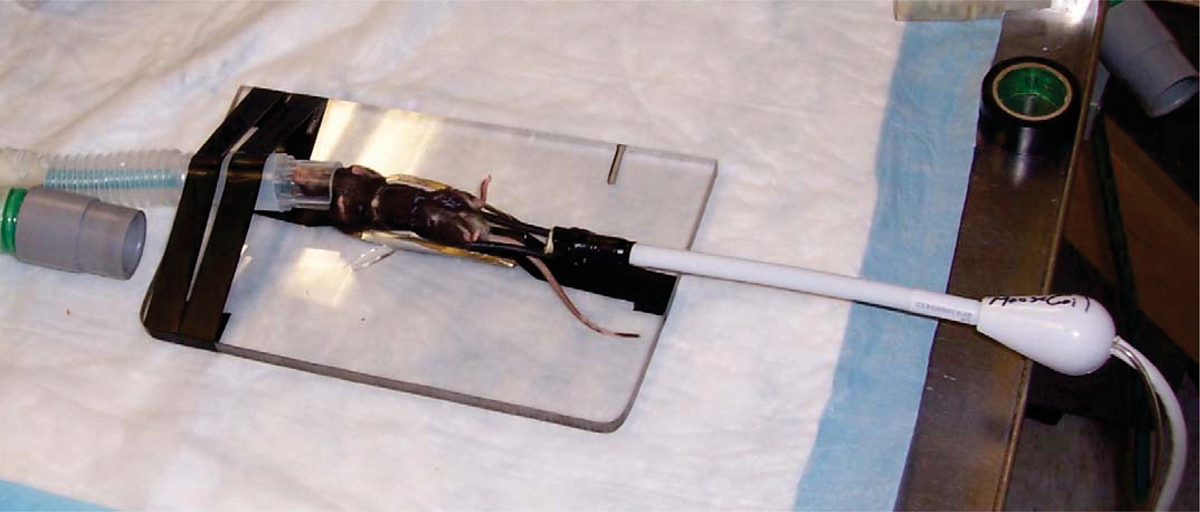
Figure 1

The magnet is a Philips 3 T Achieva/80 mt (40 × 2)/m peak, 200 mt/m/ms slew rate with Quasar Dual Gradients which is configured for standard clinical imaging. A mouse specific commercial MR coil for such a system costs in the range of $20,000–40,000 to integrate into the magnet. Instead, we used a Medrad receive only, eCoil (endorectal coil) and connector box (separate purchase for prostate imaging). This is a single loop coil with a balloon covering which is removed (coil cost = $200). The mouse is placed directly on the coil, serving as both a support structure and a close proximity receive coil (see Figure 1). A dedicated small animal ECG/respiratory gating system (SA Instruments) was used to interface with the scanner to allow for for breathing during the acquisitions. Mice were anesthetized using 0.25–0.75% isoflurane.

## Results

**Figure 2:**
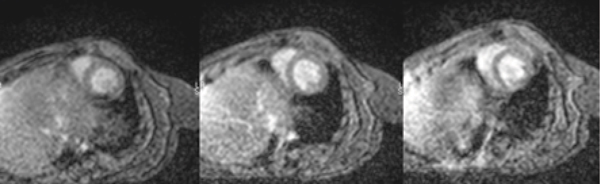
Figure 2

Short-axis ECG-gated cine gradient echo images (diastolic apical, mid and basal) are shown in Figure 2. The spatial resolution was 0.32 × 0.32 mm at a slice thickness of 1.4 mm. The temporal resolution was 24 ms at a heart rate of 300 bpm for a total of 8 true cardiac phases per R-R interval. Images were acquired for 4.0 NEX requiring 300–400 R-R intervals. Delayed inversion recovery imaging and 1^st^-pass perfusion imaging were also performed with reasonable image quality (not shown). The SNR of the blood pool to noise in figure 2 was 62.1/1 and the CNR of blood pool to myocardium was 2.45/1. The LVEF was 51.7% for the example shown.

## Conclusion

Use of a disposable single element endo coil allows the assessment of LV function in the mouse with reasonable temporal, spatial resolution and imaging characteristics without the purchase of a dedicated small animal system.

